# Towards a Stakeholder-Oriented Blockchain-Based Architecture for Electronic Health Records: Design Science Research Study

**DOI:** 10.2196/13585

**Published:** 2019-10-07

**Authors:** Jan Heinrich Beinke, Christian Fitte, Frank Teuteberg

**Affiliations:** 1 Accounting and Information Systems University of Osnabrueck Osnabrueck Germany

**Keywords:** blockchain, electronic health records, data security, information storage and retrieval

## Abstract

**Background:**

Data security issues still constitute the main reason for the sluggish dissemination of electronic health records (EHRs). Given that blockchain technology offers the possibility to verify transactions through a decentralized network, it may serve as a solution to secure health-related data. Therefore, we have identified stakeholder-specific requirements and propose a blockchain-based architecture for EHRs, while referring to the already existing scientific discussions on the potential of blockchain for use in EHRs.

**Objective:**

This study aimed to introduce blockchain technology for EHRs, based on identifying stakeholders and systematically eliciting their requirements, and to discuss the key benefits (KBs) and key challenges (KCs) of blockchain technology in the context of EHRs.

**Methods:**

The blockchain-based architecture was developed in the framework of the design science research paradigm. The requirements were identified using a structured literature review and interviews with nine health care experts. Subsequently, the proposed architecture was evaluated using 4 workshops with 15 participants.

**Results:**

We identified three major EHR stakeholder groups and 34 respective requirements. On this basis, we developed a five-layer architecture. The subsequent evaluation of the artifact was followed by the discussion of 12 KBs and 12 KCs of a blockchain-based architecture for EHRs. To address the KCs, we derived five recommendations for action for science and practice.

**Conclusions:**

Our findings indicate that blockchain technology offers considerable potential to advance EHRs. Improvements to currently available EHR solutions are expected, for instance, in the areas of data security, traceability, and automation by smart contracts. Future research could examine the patient’s acceptance of blockchain-based EHRs and cost-benefit analyses.

## Introduction

In the course of digitization, electronic health records (EHRs) have become one of the most important topics within the health care sector, as they are expected to significantly improve intersectoral collaboration and reduce health care expenses [1]. Given the fact that in many countries, including Germany, there is no government-regulated EHR system, the number of private providers on the market is rapidly increasing. However, for privacy and security concerns, many patients refrain from using an EHR because they fear that the private provider may sell their health data to make profit [2].

Similar to the health care industry, the financial sector also exchanges highly sensitive customer data. In this context, blockchain technology has recently gained attention as a possible solution to secure sensitive transactions. Blockchain technology offers potential, for instance, in the areas of disintermediation, decentralization, the reduction of necessary trust between business partners, improved protection against data manipulation, and increased automation through smart contracts [3-5]. This leads to the question, whether blockchain technology could also be able to ensure the use of EHRs. Although some authors have already analyzed the general potential of blockchain for the health care sector, a specific suggestion on how this potential can be achieved is currently missing [6]. Kuo et al, for instance, investigate the application possibilities in the field of biomedical and health care applications [7]. Although this study provides an interesting overview of the potential and challenges of blockchain-based applications, there is a lack of specific possibilities for implementation. In detail, a systematic requirement analysis for the respective field of application is missing. Further contributions present possible blockchain-based architectures for EHRs [8-10]. However, in these contributions, it often remains unclear on which scientific or practical basis the proposed architecture has been developed. Moreover, the requirements and interests of the stakeholder in the health care system are often not taken into account. In contrast to existing blockchain architectures for EHRs, our approach follows a structured scientific development and aims to include the stakeholders’ perspectives. Furthermore, there are contributions that identify the possible advantages and disadvantages of blockchain-based EHRs for different actors [6,7]. However, according to the authors, these are not complete, as not all relevant actors were investigated. Consequently, it can be stated that, to date, there is no contribution that presents a blockchain-based EHR architecture that is based on (1) multimethodically and (2) systematically collected requirements for (3) all relevant stakeholders in the health sector and also elaborates (4) the key benefits (KBs) and (5) key challenges (KCs) of the developed architecture. Similar demands for future research in this context are confirmed by other authors [7,11,12]. This motivates us to investigate the following research questions (RQs):

RQ 1: Which stakeholders have an interest in EHRs and what are their specific requirements for an EHR?

RQ 2: How can these requirements be implemented in a blockchain-based architecture?

RQ 3: Which key benefits and key challenges does the proposed blockchain-based EHR architecture provide?

The paper is structured as follows: The methodological framework is presented in the Methods section. On the basis of the design science research (DSR) method, we first identified stakeholders and collected their respective requirements using a systematic literature review and 9 interviews of health care experts. The subsequent evaluation cycle was carried out through 4 workshops with 15 participants in total, for example, health care professionals, information systems experts, and lawyers. The Results section presents the consolidated requirements of the stakeholders, the architecture, which was developed based on the identified requirements, and results from the evaluation cycles. The Discussion section focuses on the KBs and KCs that have been derived from the literature, the expert interviews, and the workshop discussions. Solutions for the identified KCs were proposed in the form of 5 recommendations for action. Limitations of the study have been elaborated, and possible further research perspectives have been pointed out. Our contribution contains interesting findings for science and practice alike, especially for EHR providers. The systematic requirement analysis can be used as a basis for the (further) development of other architectures. Besides, we have shed some light on the effects of the digital transformation on the health care sector. Another benefit of the developed architecture is that it can be prototypically implemented by companies and thus tested in a real context. The insights gained could serve for refinement of the architecture, which again allows for new findings with a different focus, such as acceptance investigations or a cost-benefit analysis.

## Methods

To answer the identified RQs, we applied the DSR paradigm that addresses human-relevant problems using innovative artifacts and simultaneously contributes new knowledge for the scientific community [13]. Designed artifacts are not only useful but also crucial to understand the problem itself. Thus, DSR is especially suitable to serve as the basis for the development of a blockchain-based EHR architecture. Although we have addressed the major security issues that hamper the diffusion of EHRs, we have involved the stakeholders in the development phase, which fosters their awareness and understanding of the technology.

**Figure 1:**
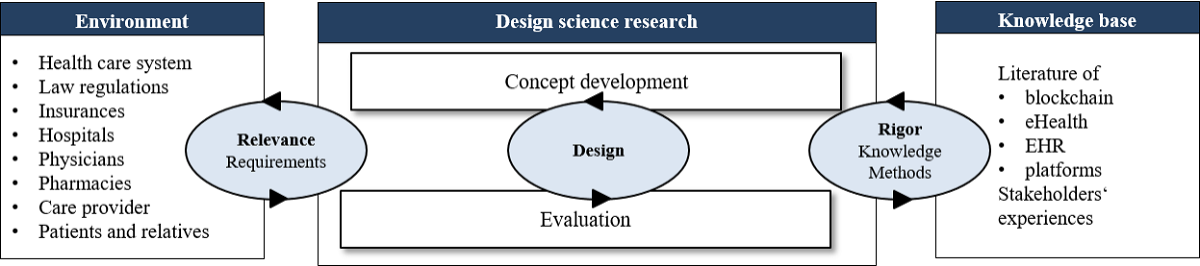
Design science research method for concept development (Hevner et al). eHealth: electronic health; EHR: electronic health record.

The development of a solution is influenced by the environment and the existing body of knowledge as visualized in Figure 1 [14]. Thereby, the relevance of the identified problems represents the connection with the environment, whereas the link to the knowledge base is represented by the recognition of results of relevant works and a rigorous application of research methods [15]. The design cycle within the DSR is altering between development and evaluation. In this study, we applied a literature review and 9 qualitative interviews to elaborate the stakeholders’ requirements. On the basis of these results, we developed a concept for a blockchain-based architecture. The intermediate results were evaluated in 4 workshops with 15 health care experts.

To identify related work, we conducted a structured literature analysis according to vom Brocke et al [16]. The search term (Blockchain OR distributed ledger) AND (EHR OR “Electronic Health Record” OR “EPR” OR “Electronic Patient Record” OR PHR OR “Personal Health Record” OR EPA OR EGA OR (Elektronische OR Digitale“ AND Patientenakte OR Gesundheitsakte)) was applied to the information systems and health care databases such as EBSCOhost, Emerald, IEEE Xplore, MEDLINE, ProQuest, PubMed, ScienceDirect, Scopus, Wiley, and Google Scholar.

**Figure 2:**
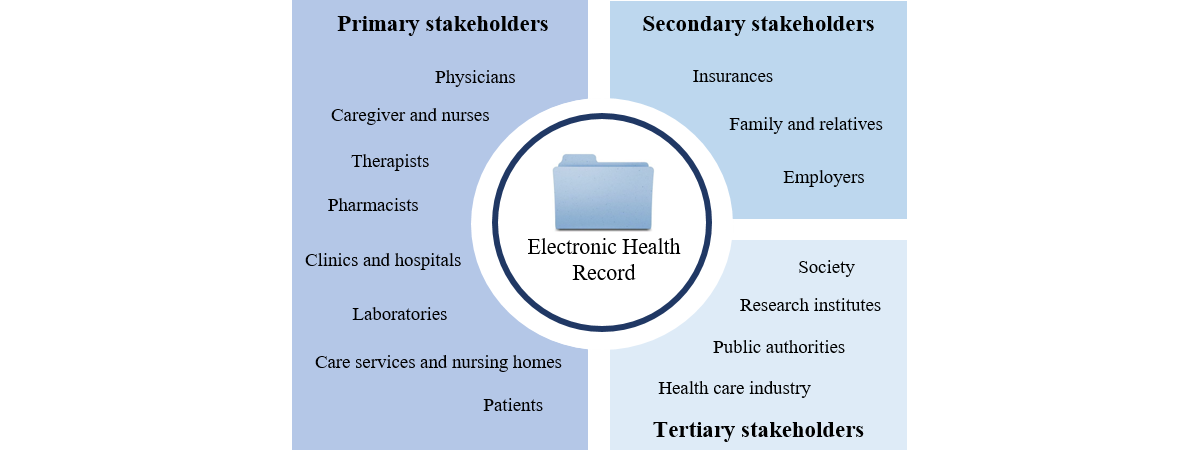
Overview of stakeholder groups.

On the basis of the outcome of our literature search, we identified all stakeholders who probably use EHRs. Although EHR providers are also key stakeholders, we excluded them from our investigation because their interest rather lies in the requirements of their customers. The remaining stakeholders can be categorized into 3 groups (Figure 2). Primary stakeholders are directly involved in providing health care, for example, physicians, caregivers and nurses, therapists, pharmacists, clinics and hospitals, laboratories, care services, nursing homes, and the patient themselves [8,17,18]. The group of secondary stakeholders includes insurances, family and relatives, and employers, whereas the tertiary stakeholder group comprises society, research institutes, public authorities, and the health care industry.

In the next phase, we collected the respective stakeholders’ requirements for EHRs from the relevant literature. To enrich the scientific perspective, we additionally conducted interviews with 9 health care experts (Table 1), who represent the mentioned stakeholder groups in the period from July to October 2018 [19]. The interviews were recorded and analyzed individually by the authors. In the Results section, we have consolidated the respective requirements. By means of the outcomes of the 4 subsequent workshops with 15 participants in total, for example, health care professionals, information system experts, and lawyers, we are in a position to evaluate our presented concept [19].

**Table 1:** Overview of interviewed experts.

No	Description	Work experience (years)	Duration (min)
E1	Pharmacist	22	40
E2	Health care consultant	2	31
E3	Pharmacist	18	27
E4	Founder and developer of an electronic health app	1	22
E5	Male nurse and case manager	30	24
E6	Health care consultant	7	36
E7	Managing director of an electronic health record	7	24
E8	Nurse	9	22
E9	Physician	10	32

## Results

### Requirements

On the basis of our systematic literature review and the 9 expert interviews, we identified a total of 34 requirements (R) for blockchain-based EHRs (Table 2). The requirements were assigned to the 3 stakeholder groups.

**Table 2:** Consolidated requirements of stakeholder groups for a blockchain-based electronic health record.

No and group		Requirement	References	E1	E2	E3	E4	E5	E6	E7	E8	E9
**Primary stakeholders**												
	R1	Data security^a^	[7,12,20]	—^b^	x	x	x	x	x	x	—	x
	R2	Data privacy^a^	[7,12,20]	x	x	x	x	x	x	x	—	x
	R3	Access/permission control, data sovereignty	[8,20-22]	x	x	—	x	—	—	x	—	x
	R4	Identity confirmation^a^	[8,20]	x	x	—	x	—	—	x	—	x
	R5	Manipulation protection/data integrity^a^	[7,21,23,24]	x	—	x	x	—	x	x	—	x
	R6	Complete health record^a^	[21]	x	x	x	x	x	—	x	x	x
	R7	Performance^a^	[7]	—	x	—	—	x	—	x	—	x
	R8	User friendly design^a^	[25]	x	x	x	x	—	—	x	x	x
	R9	Context-specific information^a^	[25]	—	x	x	—	x	x	x	x	—
	R10	Data and file storing^a^	[20]	x	x	x	x	x	x	x	—	x
	R11	Data and file sharing^a^	[8,12,20,21]	x	x	x	x	x	x	x	x	x
	R12	Interoperable and consistent data standards^a^	[12,20]	x	x	—	x	—	x	x	x	x
	R13	Intersectoral communication^a^	[12,21]	x	x	x	—	x	x	x	—	x
	R14	Ensuring trusted relationships^a^	[12,20]	—	x	—	—	—	—	x	—	
	R15	CRUD^c^ rights	[8,21]	x	—	x	—	x	—	x	x	x
	R16	Verification modus	[8,21]	x	x	—	—	—	—	x	—	x
	R17	Emergency pass	[26]	—	x	x	—	—	—	x	—	x
	R18	Medication/care plan	[27,28]	x	—	x	x	—	x	—	—	x
	R19	Tracking of state transitions^a^	[8]	x	—	x	—	x	x	x	x	x
	R20	General administrative issues	—	—	x	—	—	x	—	x	—	—
	R21	Synchronization to off-chain data^a^	[8]	—	—	—	—	—	—	—	—	—
	R22	Notification services	[8]	x	x	—	x	x	—	x	—	x
	R23	Modularity^a^	[20]	—	—	—	x	—	—	x	—	—
	R24	Patient centration	[12]	x	—	—	—	x	—	—	—	x
	R25	Workflow support^a^	[29]	x	x	—	x	—	x	x	—	—
	R26	Integration into existing systems^a^	[8,29]	x	x	x	x	—	x	—	x	x
	R27	Transfer sheet	—	—	x	x	—	x	x	x	—	x
**Secondary stakeholders**												
	R28	Scalability^a^	[7,12,20]	—	—	—	x	—	x	x	—	—
	R29	Invoice management	—	x	x	x	—	—	—	x	—	—
	R30	Modus for relatives	—	—	x	—	—	x	—	x	—	—x
**Tertiary stakeholders**												
	R31	Access to consolidated data^a^	[10]	—	x	—	—	—	—	x	—	x
	R32	Statistics	[10,30]	—	x	—	x	—	—	x	—	—
	R33	Clinical research	[31]	—	x	—	x	—	—	x	—	x
	R34	Predictive analyses	[31]	—	x	—	x	—	—	x	—	x

^a^To avoid multiple entries, requirements that apply to all stakeholder groups, for example, data security and data privacy, are listed once.

^b^Not applicable.

^c^CRUD: create, read, update, and delete.

#### Primary Stakeholders

The most important EHR concerns of this stakeholder group are connected to data security and data privacy (R1 and R2) [7,12,20]. Among other things, because of its distributed structure, the use of consensus mechanisms, and cryptographic methods, blockchain technology offers a high potential to counteract these concerns [8]. To protect the highly sensitive health data, patients should have full access and permission control and the possibility to precisely designate each actor involved (eg, physicians and relatives) [8,20,21]. Furthermore, it is essential that they retain data sovereignty, which means that the decision who has access to what data are incumbent on them (R3 and R4) [22]. For example, if patients seek a second medical opinion, they might want to avoid that the first diagnosis affects the second physician.

Nevertheless, an EHR should contain the patients’ complete treatment history to provide involved physicians with the full picture and allow for optimal treatment (R6) [21]. Thereby, it is of utmost importance that the relevant information are quickly accessible [7] but cannot be manipulated [7]. It must be ensured that these data can be updated but not manipulated [7,8,21,23]. Moreover, all stakeholder groups demand for a user-friendly design and context-specific information to avoid an information overload [25].

To enable intersectoral communication, storing, retrieving, and sharing of files and data turned out to be of high relevance (R10 and R11) [8,12,20,21]. These files and data formats should comply with consistent standards (R12) [12,20].

Furthermore, blockchain technology is capable of reducing the necessary trust between the involved actors because transactions support the aforementioned mechanisms (R14) [12,20]. In addition, blockchain technology supports data integrity, as each transaction is recorded (R5) [7]. Besides, data protection is of particular importance (R2). This can basically be supported by blockchain technology, but limitations have to be stated here. When analyzing the metadata, conclusions could be drawn about single individuals under certain circumstances [8,32]. For example, long-term monitoring of common diseases and frequently visited health care actors can provide systematic data analyses and allow for useful conclusions. However, as this conflicts with the goals of data protection, the implementation of anonymization mechanisms, such as those currently used by some cryptocurrencies as Zcash, is a suitable option for anonymizing transaction data [33].

Within this context, access control (R3) and identity confirmation (R4) again play a special role. Both could be carried out by the state, as is already the case in current health systems, such as in Estonia. Furthermore, the allocation of create, read, update, and delete (CRUD) rights must be mentioned here [8,21]. In this context, it is indispensable that specific organizational units are granted certain rights, for example, the right to insert new documents into the EHR. However, the performance of the application (R7) must be guaranteed for all transactions carried out to be attractive for the user groups [7]. The allocation or modification of, for instance, viewership rights should be tracked accordingly (R19) [8]. Before the release of data that have been newly inserted by health care professionals, the content could be checked by the respective patient by means of a *verification mode* (R16) [8,21].

Particularly, the integration of existing systems (R26; eg, hospital information system, pharmaceutical medication plan, and physicians’ patient administration system) is of importance, as this would require fewer adjustments by all actors involved [8,29]. Equally, the integration of existing workflows is requested (R25) [29]. In addition, general administrative issues should be covered, for example, the status of sick leave or the current insurance status. (R20). A continuous further development and the associated expansion of the functional scope are also planned and can be implemented in the form of individual modules (R23) [12].

Another functionality that can improve the intersectoral communication is a transfer sheet that contains all relevant information necessary for a patient’s transfer from one institution to another, for example, from a hospital in a nursing home (R27).

These transfer sheets include information on previous treatments and convey instructions to the next health care actor in charge.

In this context, there are features, such as the emergency pass, that provide all relevant emergency data (R17; eg, blood group and allergies) and a medication plan, which provides details on dosage, side effects, and drug interactions of the medications to be taken (R18) [26-28]. Notification services (R22: eg, vaccination plans) and context-specific information (R9), for example, alarm triggering when permissible vital parameters are exceeded, represent further useful enhancements [8].

Interoperability and consistent data standards (R12) as well as automation through smart contracts can also improve intersectoral communication (R13) [12,21]. However, there is often the problem that data are available in various formats and at different storage locations within the health care system, which considerably complicates a smooth communication. To enable a preferably intuitive handling for the patients, the user interface should be designed as user friendly (R8) and patient centric as possible [12,25].

#### Secondary Stakeholders

To ensure the greatest possible support from all relevant actors, the requirements set by the secondary stakeholders also have to be taken into account. For instance, it is necessary for EHR solutions to be scalable, as insurances will probably provide these to all their policyholders. Furthermore, a *relatives mode* would provide a significant added value for patients who cannot maintain files themselves (R30). In addition, health insurance companies could use the system to manage their prescriptions and monitor the compliance and efficacy of therapies.

#### Tertiary Stakeholders

For tertiary stakeholders, interesting possibilities arise from the analysis of anonymized consolidated data (R31) [10]. These data could be useful for statistical analyses about specific diseases, the efficiency of therapies (R32), and clinical research (R33) [31]. Research institutes and governments could use the data to predict diseases such as flu outbreaks (R34) [31]. However, this requires that the data set is as complete and consolidated as possible. It is, for example, also conceivable to monetize (anonymized) data. This means that institutes that seek information would have to pay the respective patients for the permission to use specific data. However, data can only be passed on actively by the user (R3, R11, and R24), and the respective institution has to clearly specify the intended use. In case of infringement, effective penalties (eg, high fines, imprisonment, or exclusion from network) could be imposed depending on the severity of the breach.

### Architecture

**Figure 3:**
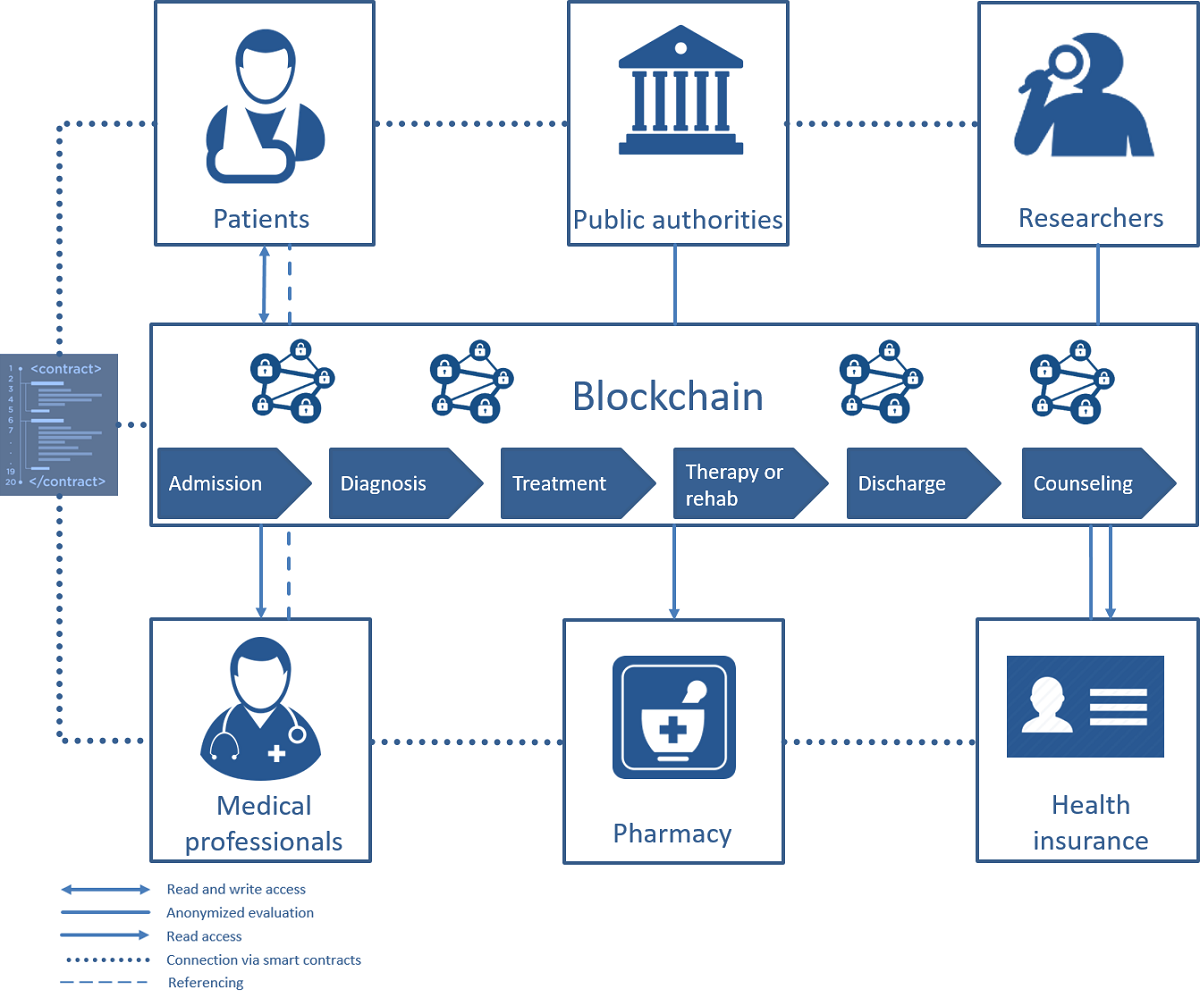
Electronic health record concept blueprint. Rehab: rehabilitation.

As part of the design science approach (first iteration), we developed an initial concept for blockchain-based EHRs within a workshop on the basis of the multimethodically collected requirements analysis. First, the identified requirements have been incorporated into the development of the concept (Figure 3).

Ølnes describes the (bitcoin) blockchain as an information infrastructure that can always be developed further [34]. In his argumentation, he refers to the definition of Hanseth and Lyytinen [35], who define the concept of information infrastructure as a common, open (and unlimited), heterogeneous, and evolving sociotechnical system consisting of a set of information technology (IT) skills and their users as well as operations and design communities. Blockchain technology offers a multitude of possible variations, for example, adding or removing actors or smart contracts (R3), using the implemented consensus mechanism. This enables that the system can be customized to meet specific needs and requirements. The developed concept (Figure 3) takes into account 3 basic options of data management: (1) it offers the possibility to interlink or reference already existing data sources; (2) it provides the possibility to store information encrypted in the blockchain; and (3) it allows to store and reference data from different data sources encrypted in the blockchain (R10 and R11). We deliberately avoided a uniform procedure to guarantee access to as many people as possible and have the highest possible flexibility. People living in countries with an appropriate digital health infrastructure are likely to choose option 1 or option 2 (R21). Although the latter offers the advantage that the data are always available, which reduces dependency on other actors, the disadvantage is the direct (encrypted) storage on the blockchain. Owing to the data protection requirements (eg, General Data Protection Regulation) and technical advantages, the authors recommend referencing the data and using the blockchain as an access management solution. This also makes it easier to adequately manage the large amount of data that are generated, for instance, during clinical studies. Patients might be notified about data changes, which they would have to approve or reject (R16).

All processes within the EHRs should be documented on the blockchain to be able to ensure complete traceability in the event of data breaches or legal disputes (R19). In a working paper for the United Research Institute for Social Development, Scott points out that blockchain-based currencies, so-called cryptocurrencies (eg, bitcoin), are a relatively simple way of managing cash holdings in countries with a weakly developed financial infrastructure and safely handling payment transactions on the spot in insecure, informal environments [36]. Similarly, the concept offers the possibility of independently maintaining the data (option 2), which is especially valuable for people who live in countries with poor (digital) health infrastructure. The access to the blockchain should be realized through a registration with an appropriately certified institution to prevent aggregation of irrelevant or trivial data (R3). Similar considerations can be found in a study by Ekblaw et al [8]. Furthermore, institutions can gain access to aggregated, anonymized data if they provide computer resources through their activities as Miner and thus ensure the network’s trustworthiness (R31). This incentive system is based on a study by Ekblaw et al [8]. The functionalities such as the access rights of the other actors to the patient data are defined by smart contracts. Automated contracts between the other players are also possible (R13, R14, and R12). In this way, the entire process can be supported, from admission and diagnosis to treatment and any associated rehabilitation to discharge and final consultation (R25).

**Figure 4:**
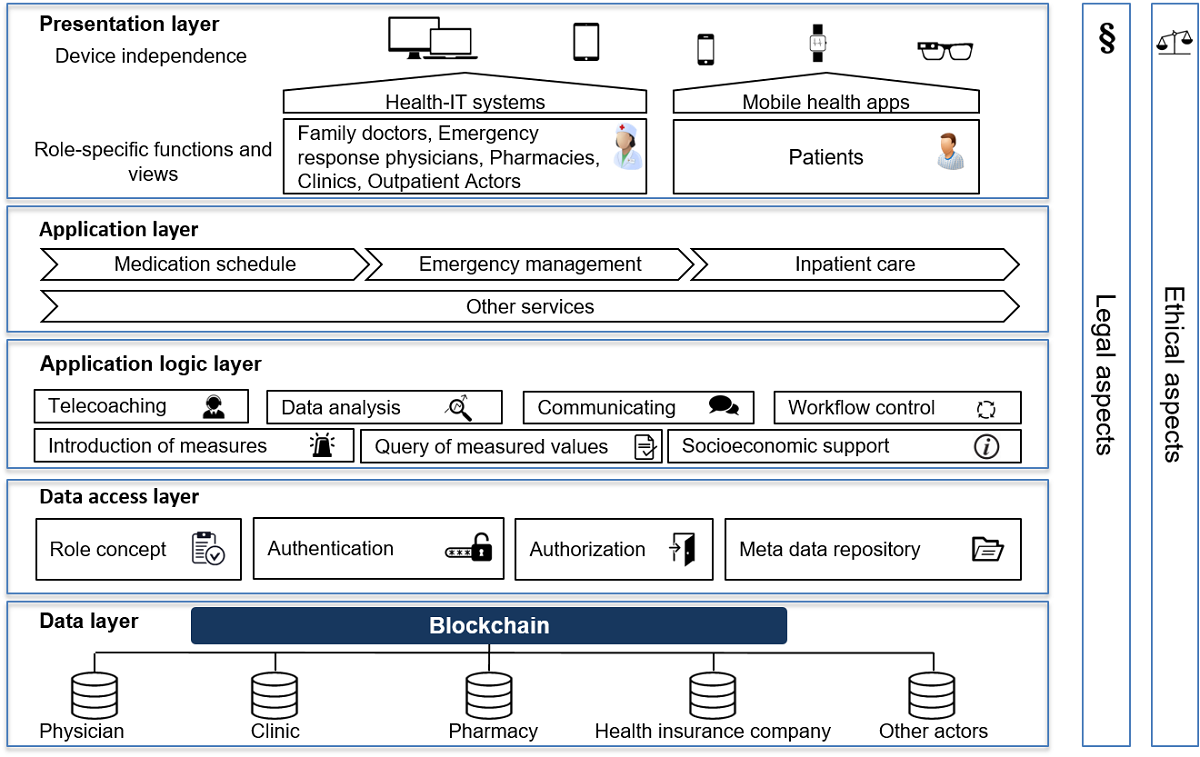
Five-tier architecture. IT: information technology.

After presenting the first concept in Figure 3 and the discussion with health care experts about the data sources and functionalities, we substantiated the concept into an n-tier architecture. The blockchain-based architecture can be divided into 5 layers as shown in Figure 4: data layer at the bottom, data access layer, application logic layer, application layer, and presentation layer. In all layers, ethical and legal implications have to be considered.

#### Data Layer

The data are standardized to ensure compatibility (R12) and intersectoral communication (R13). The integration platform provides defined communication patterns and interfaces for structured information and document exchange on the Fast Healthcare Interoperability Resources standard, the standard profiles of the Integrating the Healthcare Enterprise, the HL7 family (Health Level 7), openEHR, and the xDT family. Conformity with International Standard Organization (ISO) 21090 (health informatics — harmonized data types for information interchange) and ISO 13606 (health informatics — EHR communication) must be taken into account. For the generic description of the respective information unit, the semantics of the respective useful and information elements used are stored in the integrated metadata repository. The respective treating actor in the health sector has the possibility to provide data for the respective patient and document changes (R15 and R19).

#### Data Access Layer

Data from different sources are linked to the blockchain to ensure that the patient’s health records are as complete as possible (R8). Access control is important to ensure that the respective actors only have access to the relevant and released data (R3 and R2). In this context, the implementation of a role concept is of great importance. In addition, a precisely designed role concept in conjunction with a regulated access control renders data manipulation (R5) more difficult. Besides, a meta-data repository can support the integration and organization of relevant metadata (R26). For example, the data from the disparate systems can be better related to each other, and discrepancies, gaps, and metrics can be identified and addressed at the data structure level.

#### Application Logic Layer

The application logic layer controls the workflow of the different applications and systems (R25). Thus, data are prepared and made available for different purposes (R9 and R31). This is particularly interesting for the research area and enables various statistical evaluations (R32, R33, and R34).

#### Application Layer

On the basis of the connected data (R23), various services can be set up. A modular concept allows each stakeholder to individually configure their personal dashboards with only relevant data (R9). According to the experts surveyed, this leads, in particular, to added values for emergency passes (R17), medication plans (R18), and transfer sheets (R27). At this point, for example, the invoice management (R28) of health insurance companies can be applied. Furthermore, the opportunity to delegate administration rights to relatives could also be addressed in the form of a *relatives mode* (R30).

#### Presentation Layer

The presentation layer describes how the services are displayed to the end user. The range of functions for a user depends on his role, for example, more comprehensive functions and data are available for emergency physicians than for pharmacies. It is particularly important that the platform can be accessed either using responsive Web applications or native applications on almost all end devices and operating systems (R8). Modern smartphones, in particular, offer interesting functions with additional security measures such as fingerprint sensors and iris scanners (R1 and R3). Thus, information can also be prepared and displayed in a context-specific manner (R9). For example, different information can be displayed for a nurse wearing augmented reality glasses than for a pharmacist who receives additional information about the current medication plan and can thus be informed about drug interactions at an early stage.

### Evaluation

In our first evaluation cycle, we discussed our initial categorization of the identified stakeholders into 3 stakeholder groups (Figure 2) with 3 health care professionals and 3 information systems experts. As this categorization turned out to be too rough for the design of the EHR architecture, we refined it by splitting the stakeholders into patients, medical professionals, public authorities, pharmacies, researchers, and health insurances (Figure 3). In the second evaluation workshop with 2 health care professionals and 3 information system experts, we discussed the databases that should be included in the concept. The participants evaluated the physicians, clinics, pharmacies, and health insurance databases as most important for the EHR architecture (see data layer in Figure 4). The remaining sources were summarized in other actors.

A draft of the first concept (Figure 3) was presented to 5 health care professionals at the third evaluation workshop. Each participant added specific use cases from his profession, which we gradually included in the concept. Owing to the diversity of the use cases, we decided to begin with the 6 aspects of admission, diagnosis, treatment, therapy or rehabilitation, discharge, and counseling. In the final architecture, we reduced the complexity by implementing an application layer that did not include individual use cases but showed exemplary modules of the EHR system.

At the last evaluation workshop, we presented the final architecture (Figure 4) to 4 health care professionals, 2 lawyers, and 3 information system professionals. Additional functionalities and several data standards were discussed and incorporated into the architecture concept. Finally, we elaborated the KBs and KCs of the proposed solution.

## Discussion

### Principal Findings

After identifying the relevant stakeholders and their 34 respective requirements with the help of a literature review and expert interviews, we developed the first concept for a blockchain-based EHR. The concept was subsequently evaluated with the experts again to build a 5-tier architecture, which is presented in Figure 4. The development and introduction of blockchain-based EHRs are accompanied by several KBs and KCs. In the following, the KBs are presented before the KCs are critically discussed and possible solutions, in the form of 5 recommendations for action, are proposed.

#### Key Benefits

We identified 12 KBs of a blockchain-based EHR architecture, which we summarized with their respective sources from the literature and expert interviews in Table 3. The decentralization of the blockchain is the first KB (KB1) to be mentioned because distributed systems are usually less susceptible to system failures. Thus, there is no single point of failure (KB2). Moreover, the failure of individual nodes does not have significant effects on system security. A further advantage of the blockchain is that it has implemented various mechanisms (eg, consensus mechanism and cryptographic procedures) that render data manipulation difficult (KB3). The blockchain’s relatively high security, ensured among others by the cryptographic algorithms and decentralization, constitutes another benefit (KB4). Furthermore, the blockchain allows the tracking of entries, which makes incorrect treatment decisions traceable (KB5) and increases the patient safety. In addition, it is possible to store the entire treatment history, which significantly increases the scope of information available to the treating actors (KB6) and contributes to a general improvement in treatment quality. Smart contracts can help to automate certain processes (KB7; eg, referrals to specialists, ordering [individualized] medication). In addition, data sovereignty is firmly transferred to the patient (KB8) so that the user is the *master of his own data*. The fact that all relevant data can be made available to the corresponding actors quickly and in a standardized format, significantly increases intersectoral communication (KB9). Furthermore, it is conceivable that service providers process both invoicing and payment transactions directly using the blockchain (KB10). Possible new business models are emerging, for example, through the systematic evaluation of data or brokerage services (KB11). In summary, it can be said that the presented concept offers advantages at various levels, including technical (eg, data security), organizational (eg, intersectoral communication), and economic issues (eg, new business models). All in all, the advantages of blockchain-based EHRs could significantly improve the status quo of patient-oriented treatment (KB12).

**Table 3:** Key benefits (KB) of a blockchain-based electronic health record architecture.

No	Key benefits	References	Experts
KB1	Decentralization	[7,23]	E7
KB2	No single point of failure/vulnerability	[7]	E7
KB3	Tamper proof	[7,23,24]	E1, E2, E4, E7, E9
KB4	Data security	[23,37]	E2, E4, E7, E9
KB5	Traceability of entries	[7,23]	E1, E2
KB6	Overview of all health-related data	[21]	E7, E9
KB7	Automation by smart contracts	[38]	—^a^
KB8	Data sovereignty for patient	[7,12,23]	E1, E2, E5, E6, E7, E9
KB9	Improved intersectoral collaboration through file and data sharing	[23]	E1, E2, E3, E4, E5, E7, E8, E9
KB10	Integrated payment application	[23]	E2, E7
KB11	New mining business models for data analysis	[8,21]	E7
KB12	Patient-oriented treatment	[12]	E1, E3, E8, E9

^a^Not applicable.

#### Key Challenges

Despite all advantages, a blockchain-based EHR still faces some major challenges that are summarized with their respective sources from the literature and expert interviews in Table 4. The high energy consumption, primarily because of the use of the proof-of-work consensus mechanism, is often cited as a major challenge associated with the use of blockchain technology (KC1). This leads to high transaction costs (KC2) because of both high energy consumption and the required hardware resources (KC3). However, these challenges can primarily be addressed by 2 measures. First, other consensus mechanisms such as proof-of-stake could significantly reduce the electricity consumption; second, regulated access, in the sense of a consortium blockchain, could keep the required hardware investments manageable. Regarding access regulation, however, the question arises as to which organization is responsible (KC4). We currently consider the state or a consortium consisting of different stakeholders to be potential access regulators. Both could equally be considered when it comes to the questions of responsibility and accountability for (further) development as well as the administration of the system (KC5).

However, the fact that systematic analyses based on metadata offer comprehensive assessment possibilities and allow for conclusions, they have to be seen as a technical challenge at the same time (KC6). Although no satisfactory solution to this problem has yet been found, the cryptocurrency community is currently addressing it, for example, by means of the anonymity mechanism of the cryptocurrency Zcash [33]. Another technical aspect that needs to be addressed in the future is the so-called 51% attacks (KC7). However, they are very unlikely in a consortium. Moreover, the processing speed of blockchains is relatively slow compared with conventional databases (KC8). For example, the Bitcoin blockchain needs up to 10 min to write transactions into a new block. However, it is usually not necessary for these data to be available to other participants in real time. In addition, this time delay only affects the writing, not the reading of transaction data. The verification of imported data, which is mandatory for the highest possible data quality and timeliness (KC9), constitutes another major challenge. In principle, the respective user could confirm the entered data again before it is finally written down. However, this could also result in disadvantageous delays or users rejecting or concealing the doctor’s findings. Finally, 2 further challenges can be identified, the primary aim of which is to motivate the involved actors. On the one hand, the added value must be demonstrated to patients and health care professionals, and the necessary (technical) skills in handling such complex applications must be developed (KC10). But the benefits for the remaining stakeholders also have to be demonstrated (KC11). According to the experts, this can best be accomplished by focusing on the expected, significantly more favorable cost structure in the long term, which clearly stands out against the costs of the existing, highly fragmented, and thus relatively cost-intensive IT-system landscape. In particular, automation with smart contracts could also address a high savings potential here.

As blockchain technology is relatively new, standards are lacking, for example, regarding reference architectures and interfaces to other blockchains (KC12).

**Table 4:** Key challenges (KC) of a blockchain-based electronic health record architecture.

No	Key challenges	References	Experts
KC1	High energy consumption	[39]	—^a^
KC2	High and unpredictable transactions costs	[10,12,20,24,37]	E2, E7
KC3	Requires high storage, bandwidth and computational power, low scalability	[7,10,12,20,39,40]	E7
KC4	Access and authorization issues	[10]	E5, E6, E7
KC5	Accountability for development and administration	[23]	E5, E7
KC6	Public availability of transactions	[7,10,12,20,24,37,39,41]	E2, E3, E7, E9
KC7	51% attack	[7,37]	—
KC8	Slow processing speed	[7,12,39]	E2, E4, E7
KC9	Data imports need verification	[10,20]	E2, E4, E7, E9
KC10	Technical skills of patient and health care professional	[10]	E2, E5, E7, E8, E9
KC11	Incentives for provision of computational resources	[23]	E2, E4, E7
KC12	Standardization	[20]	E1, E3, E5, E6

^a^Not applicable.

To address these challenges, we derived 5 recommendations for action for science and practice.

#### Recommendation 1

The first recommendation for action is the development of comprehensive standards (KC12). It is conceivable to track and co-design the current standardization attempts such as ISO/TC 307 (blockchain and distributed ledger technologies) and specify this standardization for blockchain-based applications in the health sector. In addition to fundamental topics such as terminology, vulnerabilities, and reference architectures, legal issues such as the legal validity of smart contracts or governance aspects are also of interest. Standardization could thus also help to shape responsibilities for development and administration, especially in the context of IT governance (KC5).

#### Recommendation 2

The authors recommend the formation of a cross-stakeholder consortium that addresses both technical challenges (KC1, KC6, KC7, and KC11) and organizational challenges (KC4 and KC5). This consortium could establish a consortium blockchain (also called hybrid blockchain) and simplify access controls. Furthermore, consortia offer the advantage of forming a concrete entity capable of acting, which can, for example, improve the representation of interests. In principle, the question arises as to who will be involved in the consortium and how this involvement will look like. To this end, the members of the consortium should be elected from all relevant stakeholder groups.

#### Recommendation 3

Researchers and companies should continue to work on advancing blockchain technology. Thereby, encryption mechanisms must protect the data as effectively and efficiently as possible (KC6 and KC8), and consensus mechanisms must work as resource saving as possible (KC1 and KC3) without compromising security. Reducing resource use would strengthen environmental sustainability and reduce long-term financial operating costs in the form of transaction costs (KC2).

#### Recommendation 4

Costs are a decisive factor for every project. As such a project would involve high (financial) costs (KC1, KC2, KC3, and KC11), a further focus should be placed on the development of business models. Perspectives are opened here, for example, in the (anonymized) evaluation of data that can provide interesting insights for the (further) development of drugs, treatments, and therapies.

#### Recommendation 5

The 5th recommendation for action states that users must be empowered and trained to use current information and communication technologies (ICTs; KC10) and learn more about their current state of health (health literacy) to be able to trace findings to some extent and thus reduce false entries in the system (KC9). The authors are aware that probably not both goals can be realized for every user. Nevertheless, the aim should be to inform the user as well as possible about the possibilities of modern ICT and their state of health. At this point, concepts such as digital nurses or digital learning platforms could offer interesting perspectives.

### Limitations

Although the presented blockchain architecture is supposed to take all stakeholders’ requirements into account to provide optimal conditions for a qualitative health care supply, it has its limitations. First, we have not been able to interview representatives of each identified stakeholder group. For example, the specific requirements of insurance companies or research institutes need to be examined more closely. Furthermore, we did not include the opinion of the most important stakeholders in the adaption of EHR, namely the patients. The personal attitude toward technological innovations is highly subjective and depends on age, technological affinity, and pre-experiences. Therefore, quantitative surveys should be conducted to investigate the acceptance of blockchain-based EHRs. The fact that the evaluation of our results will require deep technical and organizational know-how about blockchain technology constitutes another important challenge.

### Conclusions

The aim of our analysis was to investigate whether and how blockchain technology can be used for EHRs. To do so, we applied the DSR paradigm. First, we identified 15 stakeholders and categorized them into 3 groups. With the help of a structured literature review and 9 expert interviews, we collected 34 specific requirements for EHRs (RQ1). In the next phase, we drafted the first concept for a blockchain-based architecture. Within 4 iterative evaluation cycles, we developed a 5-tier architecture that takes the identified requirements into account (RQ2). Finally, we discussed KBs and KCs of our proposed solution (RQ3) and derived 5 recommendations for action to address the KCs.

We conclude that blockchain technology offers considerable potential to improve EHRs. In contrast to currently available EHR solutions, blockchain technology offers improvements, for instance, regarding data security, traceability, and automation by smart contracts. We identified 12 KBs, which can be achieved by using blockchain technology for EHRs.

Nevertheless, there are still some KCs that need to be overcome (Table 4). Future research should address ethical, social, environmental, technological, and economic implications. First of all, research potential can be identified in the investigation of incentive programs for providing computational resources. This is connected to the question which business model would be most suitable to offer a blockchain-based EHR. In this context, cost-benefit analyses should be conducted. Furthermore, issues according to data security/privacy and the attack threat need to be analyzed. Finally, it is crucial that especially patients accept the technology. As discussed above, quantitative acceptance investigations are needed to improve applications and enhance dissemination. We expect new blockchain-based applications to emerge in the health care sector that have the potential to substantially improve existing solutions and thus the quality of health care supply.

## Abbreviations

DSR: design science research
EHR: electronic health record
ICTs: information and communication technologies
ISO: International Standard Organization
IT: information technology
KB: key benefit
KC: key challenge
RQ: research question
